# PARPs in genome stability and signal transduction: implications for cancer therapy

**DOI:** 10.1042/BST20180418

**Published:** 2018-11-12

**Authors:** Luca Palazzo, Ivan Ahel

**Affiliations:** 1 Institute of Protein Biochemistry, National Research Council, Via Pietro Castellino 111, 80131 Naples, Italy; 2 Sir William Dunn School of Pathology, University of Oxford, South Parks Road, Oxford OX1 3RE, U.K.

**Keywords:** adenosine diphosphate ribose, cancer, DNA damage response, PARPs, signalling

## Abstract

The poly(ADP-ribose) polymerase (PARP) superfamily of enzymes catalyses the ADP-ribosylation (ADPr) of target proteins by using nicotinamide adenine dinucleotide (NAD^+^) as a donor. ADPr reactions occur either in the form of attachment of a single ADP-ribose nucleotide unit on target proteins or in the form of ADP-ribose chains, with the latter called poly(ADP-ribosyl)ation. PARPs regulate many cellular processes, including the maintenance of genome stability and signal transduction. In this review, we focus on the PARP family members that possess the ability to modify proteins by poly(ADP-ribosyl)ation, namely PARP1, PARP2, Tankyrase-1, and Tankyrase-2. Here, we detail the cellular functions of PARP1 and PARP2 in the regulation of DNA damage response and describe the function of Tankyrases in Wnt-mediated signal transduction. Furthermore, we discuss how the understanding of these pathways has provided some major breakthroughs in the treatment of human cancer.

## Introduction

ADP-ribosylation (ADPr) is a post-translational modification (PTM) conserved in bacteria, viruses, and in the majority of eukaryotes [1–3]. ADPr is mainly catalysed by the ADP-ribosyltransferase (ART) superfamily of proteins, which transfer a single or multiple ADP-ribose unit(s) from nicotinamide adenine dinucleotide (NAD^+^) onto target substrates [2–4]. Substrates of ADPr can be either proteins or nucleic acids [2–4]. ARTs are widespread in different organisms and regulate diverse cellular processes as the DNA damage response (DDR), transcription, RNA metabolism and antiviral response, cell division, unfolded protein response (UPR), stress granule formation, metabolism, and cell death to cite a few [5–18].

Similarly to other PTMs, ADPr operates by altering the function/localisation/stability of targets. Additionally, ADPr acts as a scaffold for the recruitment of effector proteins (‘readers’), which are able to recognise and bind the modification through specialised protein domains, such as the macrodomain, PBZ, and WWE domains [19–23].

ADPr is a ‘reversible’ PTM; ART activity is indeed counteracted by specific hydrolases (also called ‘erasers’). Two evolutionarily unrelated protein domains are known to reverse ART's activity; catalytic macrodomains (e.g. in PARG, MacroD1, MacroD2, and TARG1), and the ARH superfamily proteins (e.g. ARH1 and ARH3) [2,23–25]. Although with possible different substrate specificities, the existence of multiple erasers of ADPr in different cellular compartments (such as the nucleus, cytosol, and membranous organelles for instance mitochondria) ensures the capability of the cells to appropriately limit and fine-tune the ADPr signal when needed [26–32]. Additionally, three classes of enzymes have been described to cleave protein ADPr in a non-canonical manner; some members of NUDIX family (such as the mammal NUDT16) [33,34], members of the Ectonucleotide pyrophosphatase/phosphodiesterase family (such as the Phosphodiesterase I found in the poison glands of rattlesnakes and the vertebrate ENPP1) [35,36], and the *Legionella pneumophila* SdeA protein [37]. Those enzymes hydrolyse the ADPr phosphodiester bond into adenosine monophosphate (AMP) and a ribose-5′-phosphate moiety linked to the substrate molecule, a protein modification known as phosphoribosylation [2,33–37].

The best-studied ART family are the poly(ADP-ribose) polymerases (PARPs), also called diphtheria toxin-like ADP-ribosyltransferases (ARTDs) [38–40]. In humans, there are 18 genes encoding PARP catalytic domain (CAT) containing proteins [38]. A slightly different classification has been proposed by Vyas et al. [41] that does not include the diverged TpT1 member (see below).

Regarding the amino acid composition of the CAT domain, PARP1 (also called ARTD1), PARP2 (ARTD2), PARP3 (ARTD3), Tankyrase-1 (PARP5a/ARTD5), and Tankyrase-2 PARP5b/ARTD6 are characterised by a histidine–tyrosine–glutamate (HYE) triad in the catalytic pocket [41]. PARP6 (ARTD17), PARP7 (ARTD14), PARP8 (ARTD16), PARP10 (ARTD10), PARP11 (ARTD11), and PARP12 (ARTD12) are characterised by a histidine–tyrosine–isoleucine (HYI) triad [41]. PARP16 (ARTD16) is characterised by a histidine–tyrosine–tyrosine (HYI) motif [41]. PARP14 (ARTD8) and PARP15 (ARTD7) contain a histidine–tyrosine–leucine (HYL) triad [41]. PARP9 (ARTD9) holds a glutamine–tyrosine–threonine (QYT) [41]. The isoform 1 of PARP13 (ARTD13) is characterised by a tyrosine–tyrosine–valine (YYV) motif, while the CAT domain is absent in the isoform 2 of PARP13 (PARP13.2) [41]. Additionally, the highly divergent TpT1 (ARTD18) is also sometimes classified as a PARP-like protein. This protein contains a histidine–histidine–valine (HHV) triad in the CAT and in yeast catalyses a NAD^+^-dependent dephosphorylation of tRNA-splicing intermediates, generating ADPr-1-phopshate through a cyclic intermediate [2,38,42]. Most of the PARPs efficiently catalyse the transfer of ADP-ribose onto proteins, albeit with different specificities. PARPs usually transfer ADP-ribose onto aspartic/glutamic acid (via ester linkages; here named as Asp/Glu-ADPr) or serine (via O-glycosylation; here named as Ser-ADPr) residues on target molecules [3,43–45].

Members of the PARP family can be also classified based on their ability to perform mono(ADP-ribosyl)ation (MARylation) or poly(ADP-ribosyl)ation (PARylation) [41]; in the latter case, the ADP-ribose units are linked together through glycosidic ribose–ribose 1″ → 2′ bonds [46]. PARPs capable of synthesis of PARylation include the DNA damage-inducible PARP1 and PARP2, which are known for their ability to produce long (up to 200 ADP-ribose units) and heterogeneous chains of branched PAR [41,46,47], and Tankyrase-1 and Tankyrase-2 [41]. It should be noted that Tankyrases synthesise PAR polymers with an average chain length of 20 units and no detectable branching [41,48]. Tankyrases are involved in multiple cellular processes, such as telomere length maintenance, mitosis, and Wnt signalling regulation [3,4]. Although initially described as able to catalyse ADP-ribose polymers up to 15-mers [49], PARP3 is currently believed to be a MARylating enzyme [3,4,38,41].

In this review, we summarise the recent advances in the understanding of PARPs by focusing on those that carry out PARylation and have functions in genome stability and signal transduction. The targeting of both cellular processes has provided promising strategies for cancer therapy. Indeed, the understanding of the cellular processes regulated by PARPs owes much to the success of PARP inhibitors in preclinical and clinical trials.

## Human PARPs

With the exception of TpT1 that appears to act as a RNA phosphotransferase, 17 human PARPs/ARTDs family members have been identified carrying a canonical ART domain [41]. PARPs are located in various cellular compartments and regulate major cellular functions, e.g. DNA damage response, transcription, chromatin structure regulation, UPR, metabolism, mitosis, telomere length maintenance, stress granule formation, antiviral response, and receptor-associated signalling [5–9,11,12,15,16,18].

The better understood PARylating PARPs/ARTDs are the DDR PARPs (PARP1 and PARP2) and Tankyrases 1 and 2 (see extensively below). Conversely, relatively little is known about the mono(ADP-ribosyl)ating (MARylating) PARPs/ARTDs and their physiological function. Recent efforts have sought to assign cellular functions to some MARylating PARPs. For instance, PARP3 is involved in the DDR and mitotic spindle assembly [50]; PARP4 (vPARP or ARTD4) has an unclear function at the mammalian vaults (ribonucleoprotein complexes) and it is possibly involved in antiviral response [51,52]; PARP6 has been proposed to have a role in cell cycle progression and has been associated with the development of colorectal cancer [53]. PARP9 possesses a unique MARylating activity specifically occurring on ubiquitin molecules and it has functions in DDR, transcription in lymphocytes, and antiviral response [52,54–57]. PARP10 is a binding protein and an inhibitor of MYC with inhibitory potential also on the NF-κB signalling pathway [58,59]. Moreover, PARP10 acts as a pro-apoptotic protein [60]. PARP11 is proposed to have a role in nuclear envelope biology [61,62], while PARP12 is a cytosolic protein but preferentially associates with the Golgi apparatus and regulates stress granule assembly, microRNA activity, and antiviral response [5,16,63]; PARP13 has been so far considered a catalytically inactive ART with functions in the assembly of stress granules [5] and in the regulation of microRNA [64], with implications in immunity and cancer [65]. PARP14 has been linked with multiple cellular functions such as survival of B-cells [66], cell migration [67], assembly of stress granules [5], transcription during inflammation processes [68], DDR [69], and antiviral response [52]. PARP15 is also involved in stress granule formation and antiviral response [5,52]. PARP16 is located at the endoplasmic reticulum and regulates UPR [8,70].

## DNA repair PARPs and signalling

The main PARPs with a direct function in the DDR are the PARylating PARP1 and PARP2, and the MARylating PARP3 [71]. PARP1 is the founding member of the PARP/ARTD family and the best-studied one. At the N-terminal end, PARP1 contains three DNA-binding zinc finger domains (ZFI: amino acid (aa) 11–89; ZFII: aa 115–199; ZFIII: aa 233–373) [46,72,73]. The central domain of PARP1 contains the BRCA1 C-terminal (BRCT) domain and the tryptophan–glycine–arginine-rich (WGR) domain [74]. At the carboxyl-terminal region, PARP1 contains the PARP homology domain, comprising of the CAT responsible for ADP-ribosylation [75–77]. PARP1, PARP2, and PARP3 share ∼60% amino acid similarity within their catalytic and WGR domains but diverge at their N-termini. PARP2 and PARP3 lack the ZF and BRCT domains and instead possess shorter unstructured N-terminal regions with poorly understood functions. In the CAT domain, PARP1, PARP2, and PARP3 share a conserved structural feature known as helical domain (HD) [75]. The crystal structure of the essential domains of PARP1 in complex with DNA double-strand breaks revealed that the HD acts as an autoinhibitory domain by blocking the access to the NAD^+^-binding site [77,78]. However, in response to the binding of PARP1 with DNA, the HD rapidly unfolds, thus allowing PARP1 catalytic activity [79]. Outside of the CAT domain is the WGR domain, which is vital for the activation of all the three damage-dependent PARPs (PARP1, PARP2, and PARP3) [74,77]. Structural studies showed that the PARP1 WGR domain makes sequence-independent contacts with both DNA backbone, near the 5′-terminus, and the HD within the CAT domain [77]. As a result of the concomitant interaction with damaged DNA and HD, the WGR transfers the information of binding to DNA to the catalytic portion of PARP1. The intramolecular contact between these two domains triggered by damaged DNA structurally destabilises the HD, leading to the catalytic activation of PARP1 [74].

PARP1 and PARP2 have been recognised as central components of the base excision repair/single-strand break repair process (BER/SSBR) [71,80]. Moreover, PARP1 and PARP2 have been found to be activated by double-strand breaks (DSBs) and required for homologous recombination (HR) at stalled or collapsed replication forks as well as for non-homologous end joining (NHEJ) [71,80].

**Figure 1.**
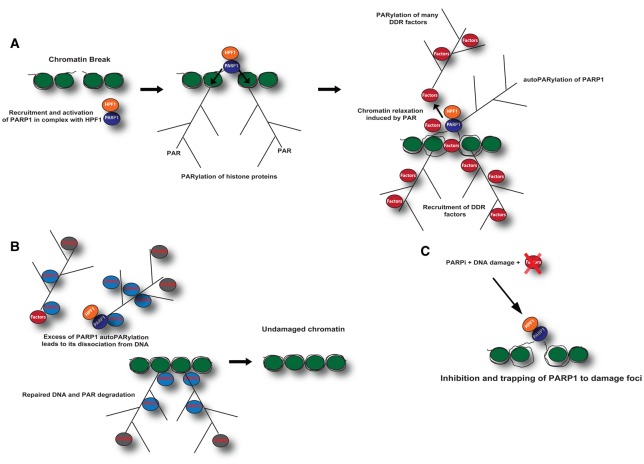
Schematic representation of PARP1-mediated DNA damage repair. (**A**) PARP1 in complex with accessory proteins, such as HPF1, is recruited to the DNA damage foci and binds damaged DNA. As a consequence of binding to damaged DNA, PARP1 PARylates serine residues on histones as well as many other factors involved in genome stability. PARylation of histone proteins allows the opening of chromatin structure and a better accessibility of DDR factors, thus facilitating DNA repair. ADP-ribosylation (ADPr) also acts as a scaffold for the recruitment of DDR effector proteins (‘readers’), which are able to recognise and bind the modification. (**B**) Upon DNA damage repair, the excess of PARP1 automodification induces the release of PARP1 from DNA damage foci and PAR chains are rapidly hydrolysed by ARH3 and PARG. (**C**) The enzymatic inhibition of PARP1 by PARP inhibitors (PARPi) results in suppression of DNA damage repair and in the trapping of PARP1 to damage foci.

Upon binding to DNA breaks, the catalytic function of PARP1 is activated to generate extensive poly(ADP-ribose) chains (PAR chains) on proteins in the proximity of DNA damage, among which include DNA repair effectors and histones [73,78,81] (Figure 1A). It has also been proposed that PARP2 binds PARP1-neosynthetised PAR chains at DNA damage sites through its unstructured N-terminal region [82]. This interaction facilitates the subsequent activation of PARP2, which seems mainly responsible for the formation of branched chains of PAR [82]. Interestingly, PARP1, PARP2, as well as PARP3 have been recently reported to directly ADP-ribosylate the DNA breaks via free phosphate groups in cellular response to DNA damage; however, how this modification affects DDR is still unclear [83–85]. Overall, the absence or the inhibition of PARP1 or PARP2 in mice or human confers sensitivity to a variety of DNA damaging agents [6,86]. The double *Parp1* and *Parp2* knockout mice show embryonic lethality suggesting significant functional redundancies between the two proteins [87].

One of the main targets of PARP1 is the histone core proteins of nucleosomes (H2A, H2B, H3, and H4) as well as the linker histone H1 [43,88–90]. Although it is not completely clear as to the mechanistic role of the highly abundant histone modification, it has been proposed that it allows the opening of chromatin structure and a better accessibility for DDR factors, thus facilitating DNA repair [91,92] (Figure 1A). The most abundant ADPr histone sites are on the specific serine resides on their tails (Ser-ADPr) [90]. Ser-ADPr fully depends on a protein called histone PARylation factor 1 (HPF1), which was identified as a key protein controlling the DNA damage-inducible PARylation of histone proteins [89,90,93] (Figure 1A). HPF1 is a PARP1-binding protein that confers specificity for substrates to PARP1, allowing specific ADPr of serine residues in histones as well as many other factors involved in genome stability [89,90]. The main target residues of ADPr in histones are Ser6 of H2B and Ser10 of H3 [43,89,90]. Unbiased mass spectrometry studies have identified a common Ser-ADPr motif in response to DNA damage. This Ser-ADPr motif is characterised by a lysine (or less frequently an arginine) residue followed by serine, which acts as an acceptor site [89]. These recent results seem to somewhat contradict previous observations, mainly describing ADPr of histones and other DDR proteins on acidic residues [94–97]. While this could be partly due to different cell lines employed and type of stress used to activate the DNA damage, the main reasons lie in the methodology applied to prepare samples and detect the modified amino acids by mass spectrometry. For instance, the majority of studies describing Asp/Glu-ADPr sites in proteins in response to oxidative insults have been conducted by employing the hydroxylamine treatment, a method that enables the sole identification of Asp and Glu modified by ADPr, therefore excluding other types of modification [94].

Importantly, PARP1 PARylates itself (autoPARylation) on multiple acceptor sites that have been characterised by MS *in vitro* and *in vivo* [29,33–36,98–101]. AutoPARylation acts as an important scaffold for recruitment of DDR factors [80]. Excessive PARP1 automodification may also induce the release of PARP1 from DNA damage foci [102,103] (Figure 1A). Extensive automodification of PARP1 is suppressed by HPF1 [93]. HPF1 also changes automodification sites in PARP1 from Asp/Glu-ADPr to Ser-ADP, although the biochemical mechanism is still unknown [89,90]. PARP1- and HPF1-dependent Ser-ADPr also occurs on many proteins involved in the maintenance of genome stability [15,100,104], for instance, the high mobility group proteins [89]. Nevertheless, many other DDR proteins may be not regulated by the PARP1-2/HPF1 complex but instead controlled by distinct PARP complexes, perhaps leading to Asp/Glu-ADPr. Indeed, the absence of HPF1 leads to a reduced ADPr of histone proteins and DNA repair factors, and conversely to the enrichment of ADPr on Asp/Glu of PARP1 itself and many other proteins [90]. Further efforts will have to clarify what the functional importance/advantage of having Ser-ADPr instead of Asp/Glu-ADPr in response to DNA damage. Deficiency of HPF1 in cells leads to sensitivity to DNA damaging drugs and PARP inhibitors (PARPi; see below) [90,93].

As noted previously, PARP1 automodification as well as the PAR chains on histone core proteins serves as a scaffold for recruitment of critical DNA repair proteins, such as XRCC1 or different chromatin remodelling proteins, to the DNA break, thus facilitating DNA repair [19,20,105–107]. The turnover of longer chains of PAR after damage largely depends on PARG hydrolase function [108–110]. In cells, PARG is inefficient in cleaving short chains of PAR and is not capable of removing MARylation [24,110,111], which are instead a substrate for ARH3 hydrolase when ADP-ribose is linked to serine residues [90,110] (Figure 1B). Glutamate-linked MARylation is hydrolysed by macrodomain-containing proteins TARG1, MacroD1 and MacroD2 [2,4,29], but is a poor substrate for ARH3 [110]. In contrast, TARG1, MacroD1, and MacroD2 are unable to hydrolyse Ser-ADPr [110].

## Modulation of DDR PAR signalling for cancer treatment

The enzymatic PARP1 inhibition by small-molecule analogues of NAD^+^ results in suppression of both SSB repair and BER and also in trapping PARP1 onto DNA lesions [112–114], which in turn causes the stalling and subsequent collapse of DNA replication forks, further resulting in replication-dependent DNA DSBs and possibly transcription conflicts [115,116]. DSBs are normally repaired by HR; however, in HR-deficient cells, such as BRCA1- and BRCA2-deficient backgrounds, lower-fidelity NHEJ occurs resulting in chromosomal aberration and ultimately cell death [117,118]. The catastrophic scenario induced by PARP inhibition could, therefore, be exploited to sensitise tumour cells to conventional treatments, such as chemotherapy or radiotherapy, which often cause DNA damage. Indeed, two groups in 2005 described the synthetic lethal (SL) interaction between PARP inhibition and BRCA1 or BRCA2 mutation suggesting a novel strategy for treating patients with BRCA mutant tumours [119–123]. Efforts in the last 20 years led to the development of many PARP inhibitors, including several that are already used in the clinics such as Veliparib (Abbvie), Rucaparib (Pfizer/Clovis), Olaparib (KuDOS/AstraZeneca), Niraparib (Merck/Tesaro) [124], Talazoparib (Lead/Biomarin/Medivation/Pfizer) [124,125], and Pamiparib (BeiGene/Merck Serono) [126,127], the latter with significant brain penetration.

All current clinically relevant PARPi are NAD^+^-mimetics and bind specifically to the PARP1 and PARP2 catalytic domain. PARPi are particularly effective (1000 times more sensitive) in the treatment of breast, ovarian, and other cancers that are BRCA1 and BRCA2 deficient [120,128–130].

Olaparib was the first PARPi approved by the U.S. Food and Drug Administration (FDA) for the treatment of patients carrying BRCA germline mutations (gBRCAm), whom have advanced ovarian cancer and already received previous lines of therapy [131,132]. The European Medicines Agency (EMA) also approved Olaparib as a maintenance treatment for BRCA mutant patients with platinum-sensitive gynecological cancers, such as high-grade serous epithelial ovarian, fallopian tube, or primary peritoneal cancers.

SL interactions between PARP inhibition and loss of function BRCA1 or BRCA2 can be expanded to sporadic tumours, a concept called ‘BRCAness’. Tumours that have not arisen from a germline gBRCAm are described to partially phenocopy the hereditary cancers in terms of HR defects [133,134]. Several mutations have been described to reproduce the phenotype of gBRCAm, such as somatically occurring mutations in either BRCA1 or BRCA2, somatic hypermethylation of BRCA1 promoter, or mutations in other genes that are involved in DSB repair and the stability of replication forks [133,134]. Beyond BRCA1 and BRCA2 mutations, BRCAness allowed to expand the SL approach to several tumours carrying deficiencies in many tumour suppressor genes involved in HR, such as ATM, ATR, PALB2, and the FANC gene family, which were shown to be sensitive to PARPi [134,135]. Notably, SL interactions between PARP inhibition and loss of function or amplification of other DDR factors, such as PALB2 and ATM, have been demonstrated by *in vitro* and xenograft studies [136–138]. Genome-wide sequencing projects revealed that somatic mutations in many other genes involved in HR occur in a wide spectrum of tumours [134], for instance, in high-grade serous ovarian cancer [139], advanced prostate cancer [140], and pancreatic cancer [141,142]. These and other cancers with HR mutations are therefore candidates for testing PARPi efficacy. Indeed, the use of Olaparib has also been approved for the treatment of metastatic, castration-resistant prostate cancer with genetic defects in DDR-encoding genes [140]. More studies will certainly expand the use of PARPi for the treatment of many other tumours, such as in acute myeloid leukaemia treatment [143]. Nevertheless, it is the obligation of the scientific community to improve the selectivity of targeted therapies. This can be achieved by an in-depth understanding of the molecular mechanisms regulated by PARPs and their cell type specificity as well as by a detailed comprehension of PARPi cytotoxic effects and by unveiling new functional interaction, such as with NEDD8/SCF and HDAC inhibitors [144,145].

A relevant topic for the clinic is the side effect of PARP inhibition. PARP1 and PARP2 have multiple important roles beyond the DDR, such as transcription, apoptosis, and immune function; the antitumour efficacy of PARPi might also reflect alterations in those functions [92]. Indeed, dose-limiting myelosuppression and central nervous system side effects were observed in some patients treated with Olaparib [146]. Being able to discriminate between the multiple cellular functions of PARP1 and PARP2 proteins, and specifically target the DDR pathway in a particular genetic background would be the next goal of the basic research-based translational research.

From a biochemical point of view, PARPi acts with different molecular mechanisms. For instance, some PARPi (such as Rucaparib, Olaparib, Niraparib, and Talazoparib) interfere with the catalytic cycle of PARP1. To a different extent, these drugs prevent PARP1 and PARP2 autoPARylation, thus trapping the enzymes on DNA lesions that they recognise [116–148] (Figure 1C); Talazoparib is ∼100 times more potent than Niraparib in trapping PARP1, which in turn traps PARP1 more potently than Olaparib and Rucaparib [147]. In contrast, Veliparib appears to have a limited ability to trap PARP1, despite its ability to inhibit PARylation [147]. These differences in trapping PARP1, rather than simply inhibiting PARylation, may be a better predictor of *in vitro* cytotoxicity in BRCA-deficient cells, with Talazoparib having the most profound cytotoxic effects [116,125,147].

Resistance to PARP inhibition is clinically relevant; multiple potential mechanisms of resistance to PARPi have been described. These include (i) silencing of DDR proteins, such as 53BP1 [149,150] or REV7 [151], which results in the reactivation of HR pathways; (ii) selective loss of PARG [152]; (iii) loss of function of proteins involved in destabilisation of the replication fork [153], such as EZH2 and MUS81 [154]; and (iv) loss of function of PARP1 itself [155]. Additionally, secondary ‘revertant’ mutations in BRCA1 or BRCA2 that restore sufficient HR function have been also described [156,157]; the same mutations are also responsible for resistance to platinum-based chemotherapy [134,156,158–160].

In the long term, the analysis of the gene expression of those DDR factors could be exploited to predict chemoresistance to PARPi. In addition, an in-depth understanding of cell type-specific PARP function is needed to address and improve patients' stratification for targeted therapies. Indeed, due to different expression of regulatory proteins and therefore with potentially different levels of regulation, the global ADPr in response to DNA damage may differ in a cell-specific manner [95]. For instance, some cell types may prefer Ser-ADPr to Asp/Glu-ADPr or vice versa, or follow alternative DDR pathways. Thus, knowing the origin of tumours and how the specific cell type respond to DNA damage induced by chemotherapy/radiotherapy may be useful to predict whether patients will be sensitive or not to PARPi.

A considerable effort has been made to identify additional SL interactions in ovarian cancers involving BRCA1 and BRCA2, which overcome PARPi resistance. The inhibition of the low-fidelity DNA polymerase-θ (Polθ) [118,161] and RAD52 [162] are synthetic lethal targets for the treatment of BRCA1-mutated cancers. These preclinical observations have led to ongoing efforts in the development of small molecule inhibiting Polθ and RAD52 [163].

In addition to PARP1 and PARP2, multiple players in ADPr regulation can be also targeted for cancer therapy, for instance, the hydrolases. The first study of breast cancer radiosensitisation by a cell-permeable specific inhibitor of PARG (PARGi; PDD00017273) [164] was reported by Gravells et al. [165]. PARGi sensitises cells to ionising radiation (IR) at the same magnitude of PARPi, although with different mechanisms of action. While PARPi radiosensitises to IR through replication-associated DNA breaks, which in turn are repaired at later times by NHEJ [121], inhibition of PARG leads to prolonged activation and persistence of PAR that results in faster DNA damage repair and rapid activation of NHEJ pathways [165]. Additionally, due to the pleiotropic functions of PARG, a severe mitotic phenotype induced by PARGi was observed, possibly due to the mitotic functions of PARG in reverting Tankyrase-mediated PARylation at the mitotic spindle (see below) [165].

The search for novel targets for cancer therapy may possibly open new research lines, perhaps looking for inhibitors of erasers and readers of ADPr (e.g. NUDT16, TARG1, ARH3, and ALC1). The advantage of targeting erasers of ADPr rather than PARPs may rely on the pleiotropic functions of catalytic enzymes, as demonstrated by the mitotic defects induced by PARGi in addition to the DNA repair ones [165]. Indeed, while each of the multiple PARPs shows a still not well-understood selectivity for certain substrates and regulates a restricted number of cellular functions, few erasers are required to act simultaneously on multiple processes and in different cellular compartments in order to revert any kind of ADPr event. Thus, compared with the inhibition of single PARPs, the inhibition of erasers of ADPr could potentially ensure the strongest impact on the overall physiology of cancer cells, thus affecting the cell survival and reducing the possibility of resistance to the treatment.

## Tankyrases

Aside from the DDR, PARPs are recognised master regulators of multiple cellular functions, such as signal transduction, cell aging, and division. In this regard, Tankyrase-1 (also called PARP5a or TNKS1 or ARTD5) and Tankyrase-2 (also called PARP5b or TNKS2 or ARTD6) are poly(ADP-ribose) polymerases with functions in telomere length maintenance [166], HR-mediated DDR [167], mitosis [168–170], and Wnt and Notch-mediated signal transduction [171,172]. Notably, Tankyrases are novel and promising targets for cancer therapy [173].

Structurally, both Tankyrases are equipped with five Ankyrin repeats (ANK), a sterile alpha motif (SAM), and CAT domain [174]. Additionally, Tankyrase-1 contains a histidine, serine, and proline-rich (HPS) region [174]. While the SAM domain is required for multimerisation of Tankyrase molecules [175,176], the ANK domain serves as a binding platform for ADPr substrates [177]. In particular, the consensus hexapeptide motif RxxPDG (where ‘x’ refers to any amino acid) within the ANK domain was shown to be necessary and sufficient for interaction with protein targets [178]. Several targets of Tankyrases were identified, among them the telomeric-repeat binding factor-1 (TRF1) [168], the insulin-responsive amino-peptidase (IRAP) [178], the 182-kDa tankyrase-binding protein (TAB182) [178], the nuclear mitotic apparatus protein-1 (NuMA1) [178], AXIN1/2 [171], 3BP2 mutated in Cherubism disease [179,180], (Bhardwaj 2017) [172], and the CBP80/CBP20-dependent translation initiation factor (CTIF) [181]. Thus, the interaction with protein substrates can be considered the specificity determinant for Tankyrases. Compared with the knowledge achieved in the understanding of PARP1 activation mechanisms, it should be noted that very little is known about the processes of catalytic activation of Tankyrases, their amino acid specificity, and the modification sites in known Tankyrase substrates (AXIN above all). Recently, new details into the activation mechanism of Tankyrases have been provided. It has been shown that intermolecular SAM–SAM contacts are required to induce polymerisation of Tankyrase molecules, in both *in vitro* and *in cellulo* [175]. Tankyrase polymerisation seems to support the catalytic activity of the enzyme [175]. Formation of Tankyrase polymers also provides a nucleation point for the assembly of non-membranous structures, named signalosomes. The advantage of the formation of signalosomes is the local concentration of proteins, which can be both enzyme and substrates, for an efficient, transient, and spatially confined process [182,183]. Unfortunately, it is still unknown which signal triggers Tankyrase polymerisation and further activation of the catalytic activity.

Notably, Tankyrase-1 and Tankyrase-2 appear to have largely overlapping functions, as deletion of either gene leads to subtle phenotypes, while double knockout of both genes is embryonic lethal [184].

**Figure 2.**
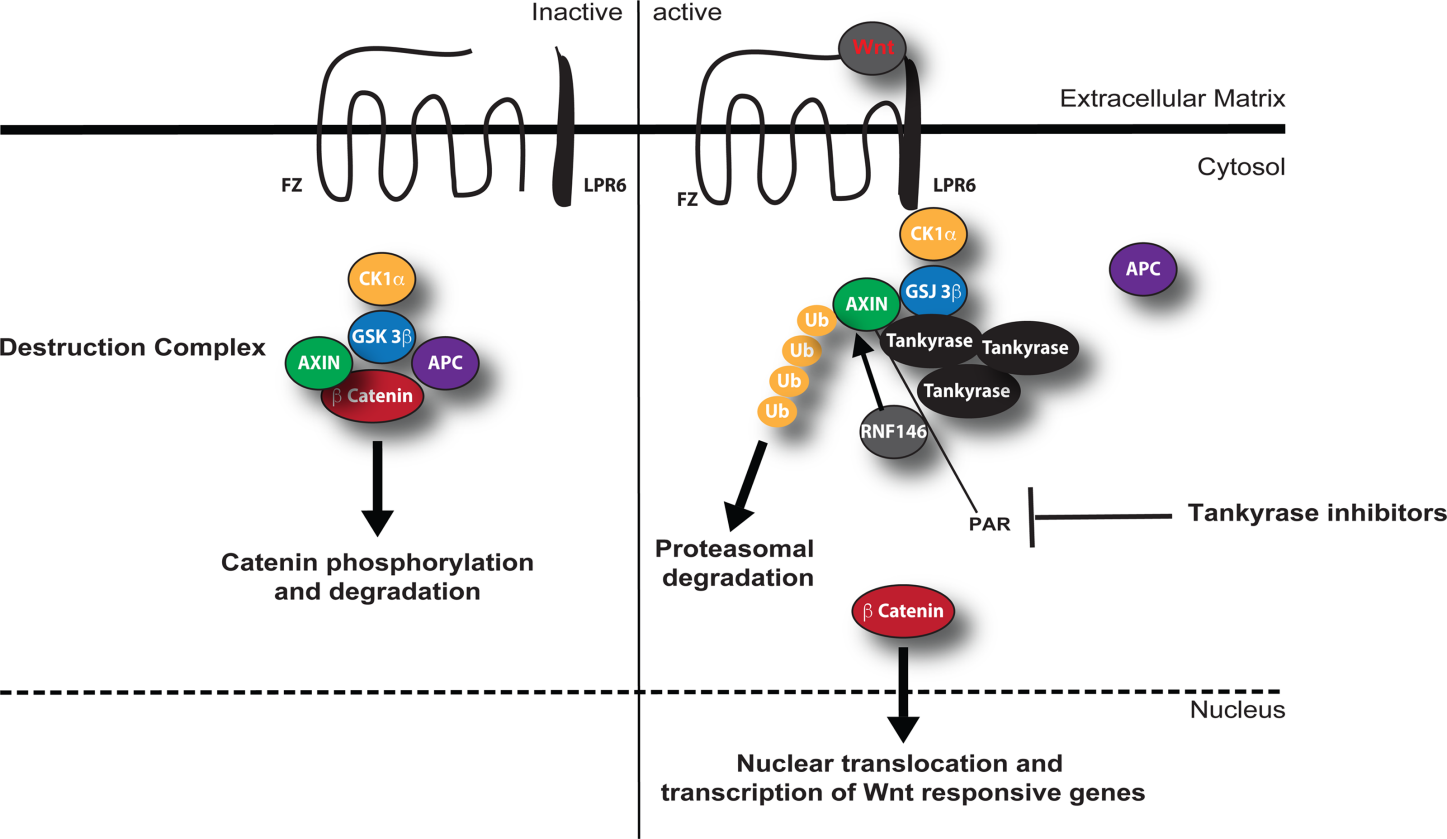
Schematic representation of Tankyrase-mediated Wnt/β-catenin signalling. The canonical Wnt signalling pathway with its major components in the inactive (left) and active (right) state.

One of the best-characterised substrates of Tankyrases is AXIN, a key regulator of the canonical Wnt signalling pathway [173,185–188] (Figure 2). In the canonical pathway, Wnt regulates the level and subcellular localisation of the β-catenin transcription factor and is thus called a β-catenin-dependent Wnt pathway. In the absence of an activating Wnt signal, glycogen synthase kinase 3β (GSK3β) collaborates with the AXIN and APC (adenomatous polyposis coli) proteins and other factors to phosphorylate β-catenin [185]. The phosphorylated β-catenin is recognised and ubiquitinated by a complex containing a β-transducin repeat-containing protein (βTrCP), then degraded by the proteasome [185] (Figure 2). Wnt binds to a seven-pass transmembrane Frizzled receptor and its co-receptor, low-density lipoprotein receptor-related protein 6 (LRP6) on the cell surface. The resulting phosphorylation cascade inhibits the AXIN/GSK3β complex and stabilises the free pools of β-catenin, which can translocate into the nucleus [185] (Figure 2). In this context, Tankyrases are required to induce AXIN degradation and in turn β-catenin stabilisation. Tankyrases can indeed bind and PARylate AXIN. Tankyrase-mediated PARylation of AXIN acts as a scaffold for the recruitment for the WWE domain-equipped E3 ubiquitin ligase RNF146, which ubiquitinates AXIN leading to its proteasomal degradation [171] (Figure 2). In the nucleus, β-catenin binds to T-cell factor (TCF) transcriptional regulators along with other cofactors and modulates transcription of various genes [185]. Mutational mechanisms activating the WNT pathway and stabilising β-catenin have been found in human cancer, especially colorectal cancer (CRC) [185–188]. Cancer mutations include inactivation of APC or AXIN or activating mutations in β-catenin, all of which lead to constitutive transcription of β-catenin/TCF-regulated genes [185–188].

## Modulation of Tankyrase signalling in cancer

The inhibition of both Tankyrases by the small NAD^+^ mimetic XAV939 was shown to inhibit growth of the APC-defective CRCs cell line by stabilising AXIN [171] (Figure 2). Tankyrase inhibitors would be presumably useful to target CRC cells that are characterised by constitutive activation of the Wnt pathway, such as those with upstream ligand-receptor defects or APC defects. In contrast, cancer cells expressing a mutant oncogenic β-catenin protein would presumably be resistant to AXIN stabilisation. Importantly, XAV939 inhibits Tankyrase-1, Tankyrase-2, PARP1, and PARP2 with comparable potency (e.g. IC_50_ values of 95, 5, 74, and 27 nM, respectively) [188]. More specific Tankyrase inhibitors were then developed with no measurable IC_50_ for PARP1 and PARP2, such as IWR-1 and IWR-2 [189–191]. Subsequently, structural studies have allowed structure-based drug design of more selective and potent Tankyrase inhibitors with cytotoxic potential demonstrated in CRC cell lines [184,192], for instance, JW74 [193], JW55 [194], WIKI4 [195,196], K-756 [197], G007-LK [198,199], and NVP-TNKS656 [200]. *In vivo* preclinical studies have demonstrated the antitumour activity of JW55 [194] and G007-LK [199] in xenograft and the *Apc*^−/−^ mouse models. Major issues reported in preclinical studies are related to the intestinal toxicity of Tankyrase inhibitors *per se* [198,201].

As discussed for inhibitors of PARP1, the multiple functions of PARPs could limit the use of such molecules only in certain clinical conditions. As mentioned, Tankyrase proteins are evolutionarily conserved and function in telomere length regulation and sister telomere cohesion, GLUT4 vesicle translocation, and possibly also mitotic spindle pole regulation [174]. Thus, targeting of Tankyrases is expected to have varied effects on cells. Notably, inhibition of Tankyrase-1 resulted in synthetic lethal effects in cells with BRCA1 or BRCA2 defects, apparently due to exacerbation of the centrosome amplification phenotype associated with BRCA deficiency [202]. Of note, both BRCA1 and Tankyrases have connected functions in the regulation of bipolar mitotic spindle formation [203]. A better understanding of the molecular complexes and additional substrates of Tankyrases as well as knowing whether the expression/function of those proteins changes in tumours originating from specific cancer cells may help patients' stratification and prognosis.

## Conclusions

In conclusion, ADPr is a central PTM regulating all the major cellular processes and thus affecting cell pathophysiology. Here, we have highlighted pertinent examples of how our understanding of ADPr signalling, gained from biochemical, structural, and cell biology studies, has been already exploited for the treatment of human cancer. Nevertheless, many other molecules linked to ADPr, thus not only ARTs, have or may have affect on cancer research; however, in many cases, their link with human pathology still needs to be uncovered.

## Abbreviations

ADPr: ADP-ribosylation
ANK: Ankyrin repeats
APC: adenomatous polyposis coli
ART: ADP-ribosyltransferase
ARTD: ADP-ribosyltransferase diphtheria toxin-like
Asp/Glu-ADPr: ADPr on aspartic/glutamic acids
BER: base excision repair
BRCT: BRCA1 C-terminal
CAT: PARP catalytic domain
CRC: colorectal cancer
DDR: DNA damage response
DSBs: double-strand breaks
gBRCAm: BRCA germline mutations
GSK3β: glycogen synthase kinase 3β
HD: helical domain
HPF1: histone PARylation factor 1
HR: homologous recombination
IR: ionising radiation
MARylating: mono(ADP-ribosyl)ating
MARylation: mono(ADP-ribosyl)ation
NAD^+^: nicotinamide adenine dinucleotide
NHEJ: non-homologous end joining
PARPs: poly(ADP-ribose) polymerases
PARylation: poly(ADP-ribosyl)ation
PTM: post-translational modification
SAM: sterile alpha motif
Ser-ADPr: ADPr on serine residues
SL: synthetic lethality
SSBR: single-strand break repair
TCF: T-cell factor
UPR: unfolded protein response.
